# Invasive stratified mucin-producing carcinoma of the cervix: a report of 34 cases of immunohistochemical and clinicopathological findings

**DOI:** 10.3389/fonc.2026.1681399

**Published:** 2026-03-02

**Authors:** Ru Wang, Linlin Zhang, Di Zhao, Ming Liu, Ting Hao, Songsheng Zhang, Lianbao Cao, Hao Yu

**Affiliations:** 1 Shandong Cancer Hospital and Institute, Shandong First Medical University and Shandong Academy of Medical Sciences, Jinan, China; 2 Obstetrics Department, Shandong Provincial Maternal and Child Health Care Hospital Affiliated to Qingdao University, Jinan, China

**Keywords:** CK5/6, CK7, p16, p40, p63, PAS, Pax-8, ISMC

## Abstract

**Objectives:**

This retrospective study aims to explore the immunohistochemical profile and clinicopathological features of invasive stratified mucin-producing carcinoma (ISMC).

**Methods:**

A total of 34 cases of ISMC were selected for this study. ISMCs are classified as either pure or mixed subtype, based on whether they are combined with tumor cells of other pathological types. We compared the clinicopathological features of ISMC with those of human papillomavirus (HPV)-associated cervical endocervical adenocarcinoma, as well as the differences between pure and mixed ISMC, through retrospective analyses.

**Results:**

Approximately 47% (16 out of 34) of the ISMC cases were classified as pure ISMC. There was no significant difference in clinicopathological features between pure ISMC and mixed ISMC. When compared to HPV-associated endocervical adenocarcinoma, ISMC cases presented with an earlier age of onset, larger tumor size, more aggressive biological behavior, and poorer prognosis. All cases exhibited block positivity for p16, positivity for CK7, and a higher Ki-67 labeling index. The expressions of CK5/6, Pax-8, P40, and P63 at the peripheral and mesenchymal junctions of the cancer foci were focally positive. Periodic acid–Schiff (PAS) staining demonstrated intra- and extracellular mucus in the cytoplasm of the tumor cells.

**Conclusion:**

The proportion of mixed ISMC cases is similar to that of pure ISMC. ISMC is more aggressive compared to HPV-associated endocervical adenocarcinoma and is associated with shorter progression-free survival. We recommend the following markers as a diagnostic panel for ISMC: p16, CK7, CK5/6, P40, P63, Pax-8, and PAS.

## Introduction

Cervical cancer is the fourth most common cancer in women worldwide and is closely linked to human papillomavirus (HPV) infection (1). Cervical cancer can be broadly classified into two categories: squamous cell carcinoma (SCC) and endocervical adenocarcinoma (ECA). However, the morbidity of ECA has been increasing annually, and the proportion of ECA has risen from 5% to almost 20% of cervical carcinoma recently (2). This may be related to the spread of cervical cancer screening, which has led to a gradual decline in the incidence of squamous cervical cancer, and improvements in medical care have led to a relative increase in the diagnosis of patients with adenocarcinoma (3, 4).

According to the International Endocervical Adenocarcinoma Criteria and Classification (IECC) system (5), ECAs are classified as human papillomavirus-associated ECA (HPV-A ECA), ECA, and human papillomavirus-independent ECA (HPV-I ECA). In 2016, Lastra et al. (6) first described invasive stratified mucin-producing carcinoma (ISMC) as a morphological variant of ECA that resembles stratified mucin-secreting intraepithelial lesions (SMILE), which is its putative predecessor (7). Previous reports indicated that ISMC exhibits considerable compositional variability, occurring either as a nanocomponent entity (pure type ISMC) or mixed with other types such as SCC, adenocarcinoma, or neuroendocrine carcinoma (mixed-type ISMC). Immunohistochemically, ISMC exhibits both squamous cell differentiation markers and glandular cell differentiation markers, reflecting its stem cell-like differentiation potential. Commonly detected markers include P16, CK5/6, P40, P63, and CK7. Previous studies employed varying criteria for identifying ISMC, with no established standard. Studies by Horn et al. (8), Park et al. (9), and Stolnicu et al. (10) indicated that ISMC exhibits greater invasiveness, with increased susceptibility to local recurrence and distant metastasis. Hodgson et al. (11) demonstrated poorer outcomes for ISMC compared to other human papillomavirus-associated usual type endocervical adenocarcinoma (HPV-A UEA). Studies conducted by Yao et al. (12) and Lee et al. (13) showed high PD-L1 positivity rates in ISMC, potentially making these patients candidates for immune checkpoint inhibitors.

However, the sample sizes of all current studies on ISMC are small, which is related to its low incidence. Therefore, we studied and analyzed 34 patients with ISMC to better understand the clinicopathological features of ISMC and to provide a reference for further diagnosis and prognosis of ISMC.

## Material and methods

### Patients

This study was conducted at Shandong Cancer Hospital and Institute (Shandong First Medical University & Shandong Academy of Medical Sciences). It is a major tertiary-care cancer center and a leading institution for oncology diagnosis, treatment, and research in Eastern China. Most patients attending the clinic are those with malignant tumors, spanning all age groups, with Asians constituting the predominant ethnic group. We collected 34 patients diagnosed with ISMC from March 2021 to May 2024 in Shandong Cancer Hospital and Institute. At the same time, we collected 70 patients diagnosed with HPV-related adenocarcinoma and 22 patients diagnosed with gastric-type endocervical adenocarcinoma (GAS) for comparison. All patients with ISMC underwent radical surgery for cervical cancer, and the pathological data used were derived from postoperative pathology. The diagnosis of the disease was made by two senior, experienced pathologists in a double-blind manner, with the assistance of a third experienced pathologist in case of disagreement. All three pathologists concurred on the present pathological data. All case information was obtained from the electronic case information system. Clinicopathological staging was performed according to the Federation of International Gynecology and Obstetrics (FIGO) staging (7th edition). Cases diagnosed by biopsy, treated by conization, or treated with neoadjuvant radiotherapy before surgery or with an ISMC component of less than 10% in the entire tumor were excluded from the study. Tumors were classified as pure if the ISMC portion made up more than 90% of the entire tumor, while mixed types were defined as the portion of ISMC accounting for 10% or more, but less than 90%, of the whole tumor (14). All patient data were collected anonymously. Informed consent was obtained from all subjects and/or their legal guardian(s). The study was approved by the Ethics Committee of the Shandong Cancer Hospital and Institute (protocol number SDTHEC202411009, dated 18 December 2024) and was conducted in accordance with the Declaration of Helsinki.

### Immunohistochemistry

All surgical resection specimens were fixed in 10% neutral formalin, routinely dehydrated, and paraffin-embedded, and 3-μm-thick serial sections were stained with hematoxylin–eosin (HE) and observed by light microscopy. Immunohistochemical staining was performed using a fully automated immunohistochemistry machine (Roche, USA) with the En Vision two-step method. Primary antibody Pax-8 was purchased from Merck (Shanghai); p16, CK7, CK5/6, p40, p63, estrogen receptors (ERs), progesterone receptors (PRs), p53, and Ki-67 were purchased from Amy Jet (Beijing). The special staining was periodic acid–Schiff (PAS) stain. Criteria for determining the results were as follows: P63, Ki-67, ER, PR, Pax8, and p40 positivity were localized in the nucleus. The Ki-67 positivity index is interpreted as a percentage count based on areas of tumor cell enrichment in the HE slices. CK7 and CK5/6 positivity were localized in the cytoplasm. The above antibody positivity criteria were defined as the presence of brown staining in the nucleus or cytoplasm, and the negative criterion was defined as staining <5%; borderline positive or patchy positive was defined as staining in 5%–25%; positive was defined as staining >25%. The p16 immunostaining pattern was interpreted as block positivity with yellow or brown granular staining in the cytoplasm or/and the nucleus. Under a 400× high-power microscope, five fields of view were randomly selected from each section, the percentage of positive cells was counted, and the results were averaged. The results were judged by the following criteria: −: staining in 0% to 5%; +: staining in 5% to 25%; ++: staining in 26% to 75%; and +++: staining in >75%. For p53, ≥80% intense nuclear staining and/or cytoplasmic staining (overexpression), no staining (complete absence), or cytoplasmic staining was defined as an aberrant pattern, and patchy weak positive nuclear staining was defined as a normal-type pattern.

### HPV genotyping

HPV subtypes were identified by quantitative real-time polymerase chain reaction (qRT-PCR). qRT-PCR was performed according to the instructions of the nucleic acid extraction kit (Jiangsu Bioperfectus Technologies Co., Ltd, China). The mixed DNA samples were put into a PCR amplifier (Roche Light cycler 96), and the results were analyzed according to the reaction Ct value using the reagent analysis software.

### Statistical analysis

For continuous variables such as age and tumor size, the component differences were analyzed by an independent-samples *t*-test or Mann–Whitney *U* test. We analyzed whether two categorical variables were independent or correlated with each other by using the *χ^2^* test or Fisher’s exact test. Differences were considered statistically significant if *p* < 0.05. Survival analysis was carried out using the Kaplan–Meier curve. Statistical analysis was performed using SPSS 2023 (IBM SPSS Statistics, version 29.0.1.0) and GraphPad Prism 10 [Version 10.4.0 (527), 23 October 2024].

## Results

### Clinical characteristics of ISMC

A total of 34 patients with ISMC were included in our study, with a mean age of onset of approximately 42 years (range, 22 to 67 years). A total of 16 (47%) patients had pure ISMC, and 18 (53%) had mixed ISMC. In mixed ISMC, seven patients were mixed with usual type endocervical adenocarcinoma (UEA), six were mixed with SCC, one was mixed with neuroendocrine carcinoma, one was mixed with mucinous adenocarcinoma, and one was mixed with both ISMC, UEA, and SCC. The maximum diameter of the tumors ranged from 1.1 to 6 cm, with a mean of approximately 3.5 cm. A total of 26 (26/34, 52.9%) patients with ISMC were detected to have had a previous infection with type 18 only, 6 patients had a previous infection with type 16 only, 2 patients had been infected by mixed type 16 HPV and type 45 HPV, 2 patients had been infected by mixed type 18 HPV and type 52 HPV, and 6 patients had been infected by mixed type 18 HPV and type 45 HPV. Thirty-two of the 34 cases of ISMC (94.1%) were of Silva pattern C, and only 2 (5.8%) of the patients were of Silva type B. Lymphovascular space invasion (LVSI) was present in 24 patients (70.6%), and lymph node metastasis (LNM) occurred in 14 patients (41.2%). Tumor infiltrating to a depth greater than 1/3 the depth of the cervical musculature was present in 29 patients (85.2%). Six patients were at the IB1 stage, 8 patients were at the IB2–IB3 stage, 2 patients were at the IIA stage, 13 patients were at the IIIC1 stage, and 1 patient was at the IVB stage. Detailed clinical information is shown in **Table 1** and **2**.

**Table 1:** Clinicopathological characteristics of ISMC.

Clinicopathological characteristics	Subtype	*N* = 34
Age, years (mean ± SD)		42.4 ± 9.4
Type		
Pure		16 (47%)
Mixed		18 (53%)
	with SCC	6
	with UEA	8
	with NEC	1
	with PA	1
	with UEA and SCC	1
	with MC	1
Maximum tumor diameter, cm (mean ± SD)		3.54 ± 1.49
HPV genotype		
	16	6 (17.6%)
	18	18 (52.9%)
	16 + 45	2 (5.8%)
	18 + 45	6 (17.6%)
	18 + 52	2 (5.8%)
Silva pattern		
	B	2 (5.8%)
	C	32 (94.1%)
Invasion depth		
	Shallow 1/3	5 (14.7%)
	Medium 1/3	9 (26.4%)
	Deep 1/3	20 (58.8%)
Lymphovascular invasion		
	Absent	10 (29.4%)
	Present	24 (70.6%)
Lymph node metastasis		
	Absent	20 (58.8%)
	Present	14 (41.2%)
FIGO stage		
	IB1	6 (17.6%)
	IB2–3	12 (35.3%)
	IIA1–2	2 (5.8%)
	IIIC1	13 (38.2%)
	IVB	1 (2.9%)

ISMC, invasive stratified mucin-producing carcinoma; SCC, squamous cell carcinoma; UEA, usual type endocervical adenocarcinoma; MC, mucinous carcinoma; NEC, neuroendocrine carcinoma; PA, papillary adenocarcinoma structure. Invasion depth, Tumor infiltrating into the mesenchyme of the uterine cervix in the inner 1/3 defined as shallow 1/3; tumor infiltrating the mesenchyme of the uterine cervix 2/3 to the ectocervix defined as deep 1/3; between shallow 1/3 and deep 1/3 defined as medium 1/3. HPV, human papillomavirus; FIGO, Federation of International Gynecology and Obstetrics.

**Table 2:** Clinical characteristics of ISMC.

No.	Age	Type	Percentage (%)	HPV	FIGO stage	Invasion depth	Tumor diameter (cm)	Silva pattern	LVSI	Invasion of nerve	Lymph node metastasis
1	41	Mixed	80%	16	IB2	Medium 1/3	2.2	C	Present	Absent	Absent
2	39	Pure		18	IIIC1	>2/3	5	C	Present	Absent	Present
3	44	Pure		18	IB2	>2/3	4	C	Present	Absent	Absent
4	44	Pure		18	IB2	>2/3	3	C	Present	Absent	Absent
5	40	Mixed	90%	18	IB1	Medium 1/3	2	C	Absent	Absent	Absent
6	33	Pure		18	IIIC1	>2/3	6	C	Present	Absent	Present
7	51	Mixed	80%	18 + 45	IB2	<1/3	3.5	C	Present	Absent	Absent
8	46	Mixed	80%	18	IIIC1	>2/3	5.5	C	Present	Absent	Present
9	56	Mixed	30%	18	IB1	>2/3	1.1	C	Present	Absent	Absent
10	67	Mixed	20%	18	IB1	<1/3	2	C	Absent	Absent	Absent
11	45	Mixed	20%	16 + 45	IB2	>2/3	2.5	C	Present	Absent	Absent
12	39	Mixed	50%	18	IB1	<1/3	1.2	C	Present	Absent	Absent
13	22	Mixed	80%	16	IIA2	>2/3	6	C	Absent	Absent	Absent
14	54	Pure		18	IB2	>2/3	3.5	B	Present	Present	Absent
15	32	Mixed	50%	18 + 45	IIIC1	Medium 1/3	1.5	B	Present	Absent	Present
16	48	Mixed	>50%	18	IIIC1	>2/3	5	C	Present	Absent	Present
17	43	Mixed	50%	16 + 45	IB2	>2/3	3	C	Absent	Absent	Absent
18	41	Pure		18 + 52	IB2	<1/3	4	C	Absent	Absent	Absent
19	46	Pure		18 + 45	IIA1	Medium 1/3	4	C	Absent	Absent	Absent
20	37	Mixed	30%	16	IB2	Medium 1/3	2	C	Present	Absent	Absent
21	61	Mixed	40%	18	IB1	Medium 1/3	1.7	C	Absent	Absent	Absent
22	37	Mixed	50%	16	IIIC1	>2/3	3.2	C	Present	Absent	Present
23	36	Mixed	70%	18	IB2	2/3	2.3	C	Absent	Absent	Absent
24	38	Pure		18	IIIC1	>2/3	3	C	Absent	Present	Present
25	39	Pure		16	IIIC1	>2/3	5	C	Present	Absent	Present
26	34	Pure		18	IIIC1	1/3	5	C	Present	Absent	Present
27	25	Pure		18 + 52	IIIC1	2/3	5.5	C	Present	Absent	Present
28	40	Pure		18	IB1	>2/3	1.7	C	Absent	Absent	Absent
29	37	Pure		18 + 45	IIIC1	2/3	6	C	Present	Absent	Present
30	36	Mixed	40%	16	IIIC1	2/3	4	C	Present	Absent	Present
31	49	Pure		18 + 45	IIIC1	>2/3	4	C	Present	Absent	Present
32	44	Pure		18	IB2	>2/3	3	C	Present	Absent	Absent
33	40	Pure		18	IB3	>2/3	5	C	Present	Absent	Absent
34	57	Mixed	30%	18 + 45	IVB	>2/3	4	C	Present	Absent	Present

ISMC, invasive stratified mucin-producing carcinoma; FIGO stage, performed according to the Federation of International Gynecology and Obstetrics (FIGO) staging 2018 (7th edition); LVSI, lymphovascular invasion.

### Immunohistochemistry characteristics of ISMC

**Figure 1:**
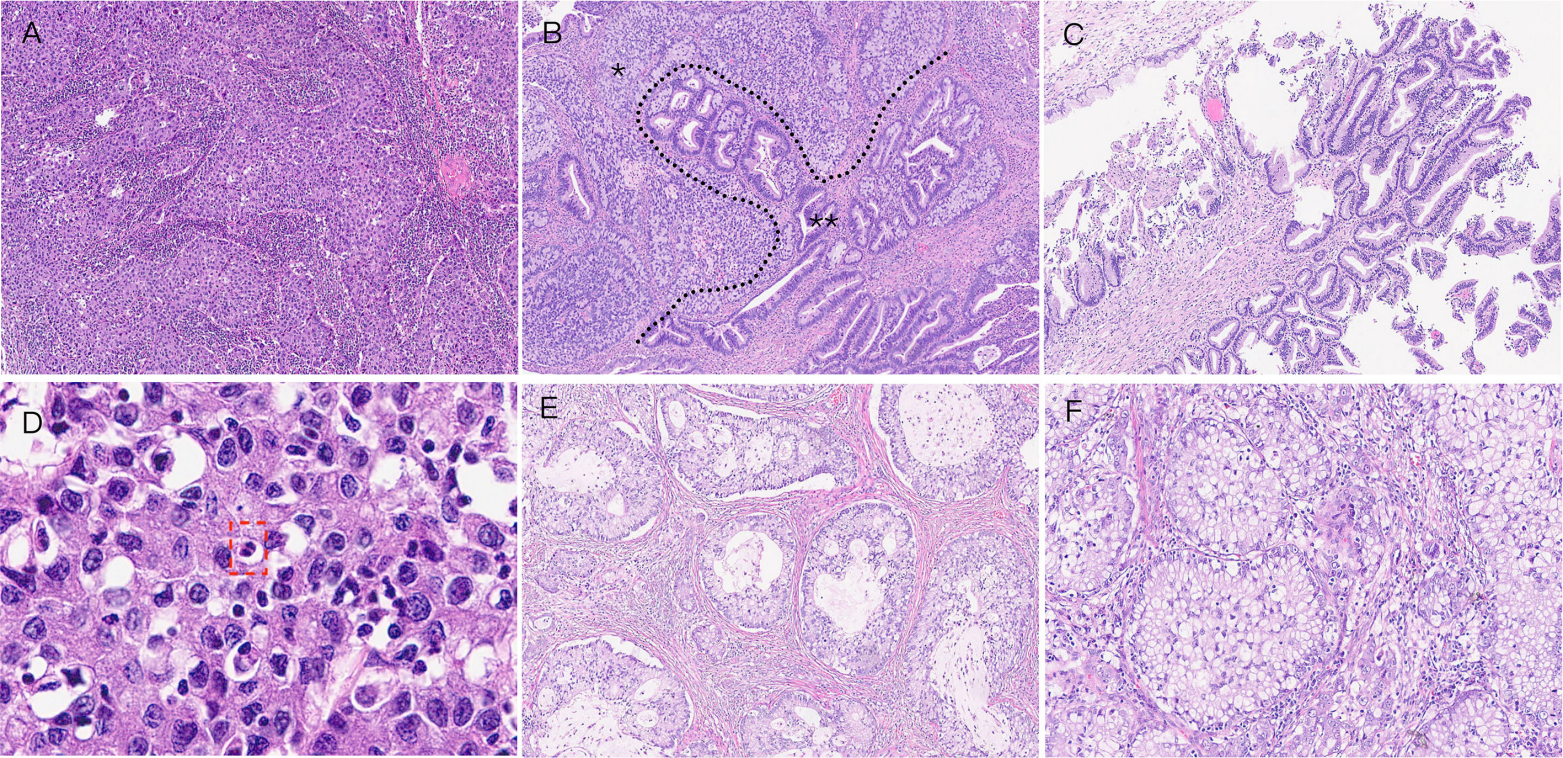
Histologic features of ISMC. **(A)** Tumor cells are typically characterized by laminar, columnar cell nests with peripheral cells arranged in a fenestrated pattern, with marked proliferation of interstitial fibrous tissue around the nests (×5). **(B)** Mixed area of both ISMC and UEA components (×5). **(C)** Fluffy (×5). **(D)** Tumor cell cytoplasm is eosinophilic and nuclear schizophrenia, singular and polymorphic nucleus seen (×20). **(E)** Lumen-forming (×5). **(F)** Mucous cell-rich type (×10).

**Figure 2:**
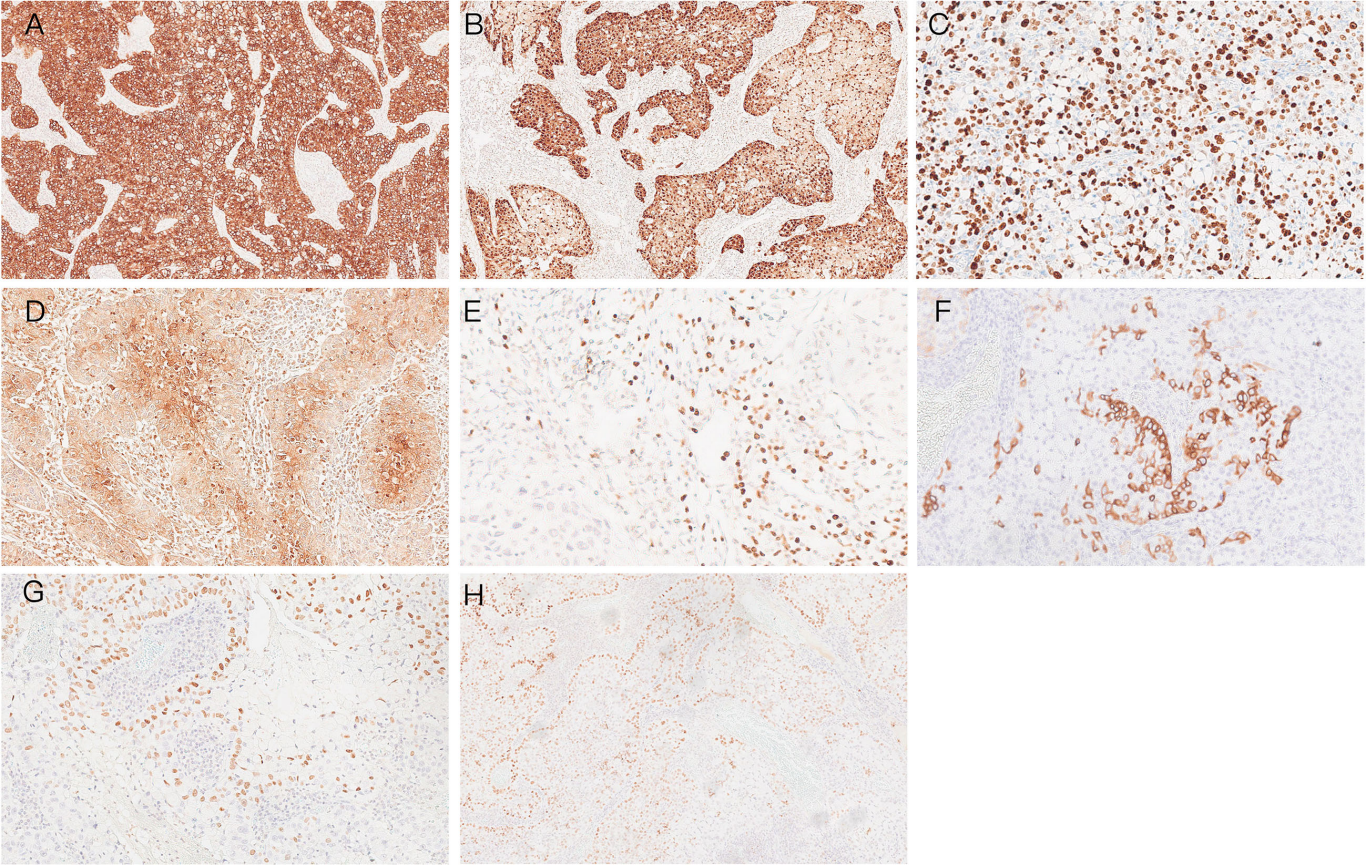
Immunohistochemistry characteristics of ISMC. **(A)** CK7 strongly positive (×5). **(B)** P16 strongly positive (×5). **(C)** Ki-67 positivity index approximately 80% (×10). **(D)** CEA positive (×10). **(E)** Pax-8 focal positive (×20). **(F)** CK5/6 focal positive (×10). **(G)** P40 focal positive (×10). **(H)** P63 focal positive (×5).

All the tumors showed block-like positivity for p16 and positivity for CK7 and PAS staining. The Ki-67 index of the tumors was between 20% and 90% (71.8% ± 12%, *n* = 26). They were always negative for CK5/6, P63, P40, or Pax-8, except for a few cases that showed borderline positive. All the tumors were negative for ER and PR (**Table 3**; **Figures 1**, **2**).

**Table 3:** Immunohistochemistry characteristics of ISMC.

No	p16	Ki-67	p53	ER	PR	CK7	Pax-8	CK5/6	p63	P40	PAS
1	+++	70%	NA	−	−	+++	Borderline+	−	−	−	+
2	+++	NA	NA	−	−	+++	−	−	−	−	+
3	+++	80%	NA	−	−	+++	−	−	NA	NA	+
4	+++	80%	Normal	NA	NA	+++	−	−	Borderline+	Borderline+	NA
5	+++	70%	NA	−	−	+++	NA	−	−	−	+
6	+++	NA	Normal	NA	NA	+++	−	+	NA	NA	NA
7	+++	NA	Normal	−	−	+++	−	−	−	−	+
8	+++	60%	Normal	−	−	+++	−	NA	−	−	NA
9	+++	80%	NA	−	−	+++	−	NA	−	−	NA
10	+++	80%	NA	−	−	+++	NA	+	Borderline+	Borderline+	+
11	+++	70%	NA	−	−	+++	−	−	−	−	+
12	+++	NA	Normal	−	−	+++	−	−	−	−	+
13	+++	60%	NA	−	−	+++	NA	−	NA	NA	NA
14	+++	80%	Normal	NA	NA	+++	−	−	−	−	NA
15	+++	80%	Normal	−	−	+++	NA	+	−	−	+
16	+++	NA	NA	−	−	+++	−	−	NA	NA	+
17	+++	60%	Normal	NA	NA	+++	−	−	−	−	NA
18	+++	30%	Normal	−	−	+++	−	−	−	−	+
19	+++	90%	NA	−	−	+++	NA	NA	−	−	+
20	+++	NA	NA	−	−	+++	−	+	Borderline+	Borderline+	NA
21	+++	20%	Normal	−	−	+++	−	−	+	+	+
22	+++	60%	NA	−	−	+++	NA	−	Borderline+	Borderline+	+
23	+++	NA	NA	−	−	++	−	−	+	+	+
24	+++	80%	Normal	−	−	+++	NA	−	−	−	+
25	+++	90%	Normal	−	−	+++	NA	−	−	−	+
26	+++	80%	Normal	−	−	+++	NA	−	−	−	NA
27	+++	70%	Normal	−	−	+++	+	−	NA	NA	+
28	+++	NA	Normal	NA	NA	+++	Borderline+	−	−	−	+
29	+++	50%	NA	−	−	+++	−	Borderline+	−	−	NA
30	+++	60%	NA	−	−	+++	−	−	Borderline+	Borderline+	+
31	+++	70%	Normal	−	−	+++	NA	−	−	−	+
32	+++	50%	NA	NA	NA	+++	−	−	−	−	+
33	+++	70%	NA	−	−	+++	NA	Borderline+	−	−	NA
34	+++	70%	Normal	−	−	+++	−	−	Borderline+	Borderline+	+

ISMC, invasive stratified mucin-producing carcinoma; NA, not available; (, negative; +, positive; Borderline+, staining in 5%–25%; +++, staining in > 75%.

### Comparison of clinicopathological features of HPV-A UEA

Patients with ISMC had an earlier mean age of onset than HPV-A UEA (42.4 ± 9.4 vs. 48.7 ± 10.2, *p* = 0.003) and a larger mean tumor diameter (3.541 ± 1.487 vs. 2.303 ± 1.499, *p* < 0.001). ISMC invaded deeper into the cervical muscular layer (*p* = 0.006), with a higher frequency of LVSI (*p* < 0.001) and LNM (*p* < 0.001), a later FIGO stage (*p* < 0.001), and worse Silva staging (*p* = 0.002). Detailed clinicopathological information is shown in **Table 4**.

**Table 4:** Comparison of clinicopathological features between HPV-A UEA and ISMC, pure ISMC, and mixed ISMC.

Variables	ISMC (*N* = 34)	HPV-A UEA (*N* = 70)	*p*-value	Pure ISMC (*N* = 16)	Mixed ISMC (*N* = 18)	*p*-value
Age, years	42.4 ± 9.4	48.7 ± 10.2	0.003	40.56 ± 6.74	44.00 ± 11.17	0.293
Maximum tumor diameter (cm)	3.541 ± 1.487	2.303 ± 1.499	<0.001	4.05 ± 1.29	3.08 ± 1.53	0.059
Invasion depth			0.007			1.000
Shallow 1/3	5	29		2	3	
Deep 2/3	29	41		14	15	
LVSI			<0.001			0.715
No	10	45		4	6	
Present	24	25		12	12	
LNM			0.002			1.000
No	20	60		9	11	
Present	14	10		7	7	
FIGO stage 2018			0.004			1.000
I stage	18	56		8	10	
≥II stage	16	14		8	8	
Silva pattern			<0.001			1.000
A+B	2	30		1	1	
C	32	40		15	17	

ISMC, invasive stratified mucin-producing carcinoma; HPV-A UEA, human papillomavirus-associated usual type endocervical adenocarcinoma; LVSI, lymphovascular invasion; LNM, lymph node metastasis; Invasion depth, Tumor infiltrating into the mesenchyme of the uterine cervix in the inner 1/3 defined as shallow 1/3; tumor infiltrating the mesenchyme of the uterine cervix 2/3 to the ectocervix defined as deep 1/3; between shallow 1/3 and deep 1/3 defined as medium 1/3.

For age, normally distributed with homogeneity of variance, an independent samples *t*-test was employed to compare the two groups. For maximum tumor diameter, non-normally distributed with homogeneity of variance, the Mann–Whitney *U* test was employed to compare the two groups. For categorical variables, if the sample size *n* < 40 or *T* < 5, Fisher’s exact test shall be employed; if *n* ≥ 40, *T* ≥ 5, the chi-square test shall be used. Differences were considered statistically significant if bilateral *p*-value < 0.05.

### Comparison of clinicopathological features of pure and mixed ISMC

As shown in **Table 4**, pure ISMC showed no significant difference in age of onset, tumor size, LVSI, LNM, FIGO stage, or Silva pattern compared to mixed ISMC (*p* > 0.05) (**Table 4**).

### Survival information of patients with ISMC

**Figure 3:**
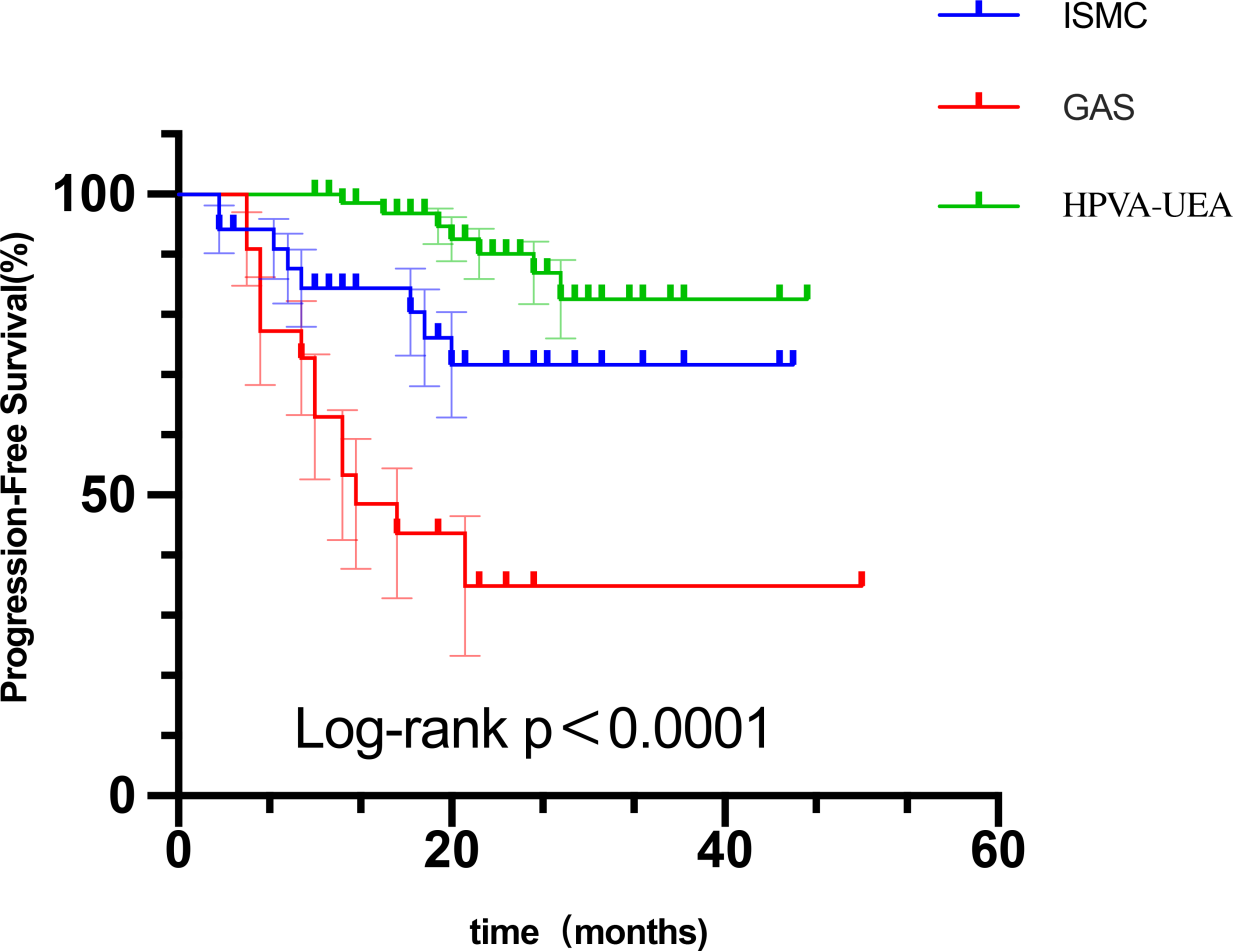
Kaplan–Meier curve showing progression-free survival in ISMC and HPV-A-UEA and gastric-type endocervical adenocarcinoma (GAS).

Follow-up ranged from 3 to 45 months; 3 patients were lost to follow-up, and 7 of the remaining patients had tumor metastasis after treatment. The most common site was lymph node systemic metastasis (6/7), including pelvic lymph nodes, retroperitoneal lymph nodes, mediastinal lymph nodes, and supraclavicular lymph nodes; two of these patients had recurrence of the vaginal dissection. One patient had lung metastasis. To better understand the survival prognosis of patients with ISMC, we compared and analyzed the survival data of patients with ISMC with those of 70 cases of HPV-A UEA and 22 cases of gastric-type adenocarcinoma during the same period of time, and the results showed that the prognosis of ISMC was worse than that of HPV-A UEA (*p* = 0.0321) and better than that of gastric-type adenocarcinoma (*p* = 0.0109) (**Figure 3**).

## Discussion

SMILE, initially described by Park et al. in 2000, is a pre-invasive tumor of the uterine cervix that is believed to originate from reservoir cells in the transformation zone (7). These cervical reserve cells, found at the squamous–columnar junction of the cervix, are a type of pluripotent stem cell capable of differentiating into both squamous and columnar cells. They play a crucial role in the repair and proliferation of cervical epithelial tissue. However, they are also the primary cells responsible for the development of cervical cancer and precancerous lesions, particularly following high-risk HPV infection.

The World Health Organization (WHO) classified SMILE as HPV-associated adenocarcinoma *in situ* of the cervix in its classification of cervical tumors of the female reproductive system in 2020. Previous studies indicated that approximately 58% of patients with ISMC have been infected with HPV (15), but in our study, approximately 76% (26 out of 34) of patients tested positive for HPV18. The pathomorphological features of SMILE overlap with those of high-grade squamous intraepithelial lesions (HSIL) and typical adenocarcinoma *in situ*. The tumor cells are layered and exhibit a certain degree of cellular atypia, similar to HSIL; however, SMILE tumor cells contain prominent intracellular mucus, which distinguishes them from HSIL. Additionally, SMILE differs from typical adenocarcinoma *in situ*, which displays a distinct glandular cavity structure, whereas SMILE does not form glandular luminal structures. In SMILE, varying numbers of mitotic figures and apoptotic bodies can be observed, setting it apart from benign glandular hyperplasia. Furthermore, SMILE often coexists with HSIL and adenocarcinoma *in situ*, and it rarely occurs in isolation. ISMC is characterized by infiltrative destructive growth and progresses from SMILE (7, 15–17). We can observe that a small number of patients show both SMILE and ISMC, indicating a gradual transition from SMILE to ISMC. This phenomenon suggests that ISMC is likely to develop from SMILE. However, this transition is not seen in every case of ISMC.

Since its definition in 2016, the morphological features of ISMC have been progressively described. ISMC tumor cells are characterized by their infiltration into the cervical mesenchyme, forming nests of complex columnar epithelial cells. These columnar-shaped tumor cells exhibit moderately heterogeneous nuclei, mitotic figures, and apoptotic vesicles. The peripheral cells are fenestrated, and varying amounts of intracellular mucus are stratified throughout the tissue thickness, with no clear gland formation (5, 6). Because ISMC often coexists with adenocarcinoma or SCC of the cervix and can be difficult to identify when poorly differentiated, cases were previously misdiagnosed as adenosquamous carcinoma before the ISMC entity was recognized in 2020. The Ki-67 index typically ranges between 20% and 90%, with an average of 71.8% ± 12% (*n* = 26), while p53 is nearly wild type. ERs and PRs are negative. P16 immunostaining positivity is frequently associated with high-risk HPV infection; our study indicated that P16 was positive in 34 cases of ISMC. CK7 is a specific marker for glandular epithelial cell lineage, and we found CK7 immunostaining to be positive in all ISMC cases. In contrast, P40 and p63 immunostaining were predominantly negative or only locally positive in 21 out of 29 and 6 out of 29 cases, respectively. Cervical reserve cells may express P40 and p63 when they show a basal-like cell differentiation pattern arranged in a fence-like structure, particularly with p63 expression. PAS staining reveals an abundance of mucin within the cytoplasm of ISMC. Adenoid basal carcinoma consistently displays a nest-like structure with palisading around the nests but lacks mucin-producing cells and shows strong expression of p63 (18). Pax-8 is primarily expressed in serous and endometrial carcinomas, while it is absent in mucinous carcinomas like ISMC. This suggests that PAX-8 may be linked to its origin from reserve cells. Cytokeratin 5/6 (CK5/6) is mainly expressed in squamous epithelium. In pure ISMC, CK5/6 is negative; however, in ISMC mixed with squamous carcinoma, CK5/6 can be locally positive in the squamous carcinoma cells. We recommend using a diagnostic panel that includes P16, CK7, CK5/6, P40, p63, Pax-8, and PAS immunostaining for ISMC diagnosis. This panel can help distinguish ISMC from adenosquamous carcinoma, mucinous adenocarcinoma, adenoid basal carcinoma, serous carcinoma, and endometrial carcinoma (6, 9, 19–21).

According to recent results, almost half of all ISMCs were mixed. The most common mixed types are UEA, SCC, and mucinous carcinoma (5, 6, 12). In our study, some individual ISMCs can be mixed with small cell neuroendocrine carcinoma. On the other hand, because of its mixed morphology, some researchers consider it a variant of adenosquamous carcinoma (22). Based on previous research, ISMC accounted for 10% of ECA (9, 12, 14); the exact incidence had not been reported.

Both SMILE and ISMC are believed to originate from reservoir cells located in the transformation zone. SOX2 is a marker for stem cells, thought to arise from cervical reserve cells. Park et al. demonstrated that ISMC can express stemness markers associated with reserve cells to varying degrees (5, 23). During the transdifferentiation process, epithelial cells gain the ability to migrate and invade, a phenomenon known as epithelial–mesenchymal transition (EMT). EMT plays a crucial role in tumor metastasis and recurrence. Park et al. showed that ISMC exhibits a high level of expression of Snail and Twist, which are two major transcription factors related to EMT. These factors regulate EMT by downregulating E-cadherin or interacting with other transcription factors. This suggests that ISMC may demonstrate more aggressive biological behavior and a poorer prognosis, including advanced FIGO stage, LNM, and Silva pattern C (8, 24, 25).

Sasieni et al. (4) showed that the mean age of onset of adenocarcinoma was 3.6 years younger than that of squamous carcinoma in patients with FIGO stage IB+ cervical cancer. Our study further analyzed the mean age of onset of disease in patients with ISMC in the HPV-A UEA subgroup, which was 3.5 years earlier than in HPV-A-UEA. Stolnicu et al. (10) and Yao et al. (12) reported that pure ISMC tumors were larger in size compared to the mixed type. Stolnicu et al. demonstrated that pure syngeneic ISMC was more malignant and had a worse prognosis than the mixed type, with a higher rate of LVSI, LNM, and pelvic recurrence. However, the results of our study were consistent with those of Yao et al., with no significant difference (*p* > 0.05). Studies with a larger sample size are needed to confirm this aspect.

Our study showed that ISMC is characterized by larger tumor diameters, an earlier age of onset, and a higher incidence of LVSI and LNM compared to HPV-associated endocervical adenocarcinoma (HPV-A UEA). The majority of ISMC cases displayed the Silva C pattern (32 out of 34), consistent with numerous previous studies, highlighting the highly invasive nature of ISMC and its associated poor prognosis. In the colorectum, ISMC is recognized as a high-grade carcinoma that can develop into signet ring cell carcinoma as the tumor progresses (26). Furthermore, ISMC exhibits more aggressive biological behavior and a worse prognosis than both UEA and SCC. It is also more likely to metastasize to lymph nodes and distant sites, particularly the lungs (8).

Park et al., Yao et al., and Choi et al. demonstrated that ISMC harbored pathogenic (likely) mutations in several genes, including STK11, MET, FANCA, PALB2, PIK3CA, ERBB2, TP53, and PTEN (9, 12, 27). These mutations have also been observed in other cervical carcinomas and may be relevant to the invasive biological behavior of ISMC (28–30). The STK11 mutation is associated with the autosomal dominant genetic disorder Peutz–Jeghers syndrome. In cervical cancer, STK11 mutations correlate with poorer clinical outcomes and are frequently reported in gastric-type adenocarcinoma, an HPV-independent subtype that demonstrates aggressive behavior; however, patients with these mutations do not typically present with Peutz–Jeghers syndrome. Inactivating mutations in both the PIK3CA and STK11 genes can activate the PI3K/AKT/mTOR signaling pathway. Therefore, mTOR inhibitors may prove to be a significant therapeutic option for patients with ISMC with such mutations. Research by Zammataro et al. indicated that the use of PIK3CA inhibitors can significantly reduce cervical cancer lesions in mice, suggesting that targeted therapy may be applicable for patients with PIK3CA-mutated ISMC (31).

Previous researchers reported that 62.5% to 100% of patients with ISMC exhibited PD-L1 positivity (CPS ≥ 1) (12, 13, 27, 32). This finding implies that the overexpression of PD-L1 may play a role in the aggressive behavior of ISMCs and is associated with a poorer prognosis. Given that the anti-PD-1 inhibitor pembrolizumab has been approved for use in treating advanced cervical cancer patients who have Food and Drug Administration (FDA)-validated PD-L1 positivity (CPS ≥ 1) (33), patients with ISMC could potentially benefit from PD-1/PD-L1 immunotherapy (12). In a study conducted by Cho et al., it was found that the prognosis for ISMC is worse than that for HPV-A UEA, but better than that for HPV-I ECA, including clear cell adenocarcinoma and gastric adenocarcinoma. This aligns with our findings (34). Additionally, Feng et al. performed a genomic analysis on eight ISMC samples using whole-exome sequencing. All samples exhibited a low tumor mutation burden (TMB) and were classified as microsatellite stable (MSS). Notably, MUC4 mutations were found to be specific to pure type ISMCs, while mutations in DMD and DMKN were predominant in mixed ISMCs (32).

This study was conducted at a single center, which may limit the generalizability of the findings. The patient cohort, along with the diagnostic and therapeutic protocols, could have been influenced by the specific medical conditions, geographical characteristics, and clinical practice patterns of that center. Additionally, the sample size was small and not very representative, which constrained subgroup analyses. This limitation may have made it challenging to identify genuine differences between groups and increased the risk of sampling error. During data collection, there may have been selection bias, such as subjectivity in inclusion and exclusion criteria, as well as information bias due to missing data or measurement errors. These factors could have impacted the findings. Furthermore, the study covers a significant time frame during which clinical guidelines and techniques have evolved, introducing potential confounding variables. To confirm the reliability and generalizability of these findings, future research should involve multicenter, large-scale prospective studies.

In conclusion, the prevalence of mixed ISMC is similar to the pure type, but it is more aggressive than HPV-A UEA, resulting in a poorer prognosis. Immunohistochemical analysis shows P16 blockage, positive CK7 and PAS staining, and a high Ki-67 index. Additionally, CK5/6, Pax-8, P40, and P63 are focally positive in the cancer foci’s peripheral and mesenchymal junctions. Future studies with larger sample sizes and genetic analyses are essential to enhance our understanding and improve patient outcomes.

## Data Availability

The raw data supporting the conclusions of this article will be made available by the authors, without undue reservation.
