# Supplementary material for: Invasive stratified mucin-producing carcinoma of the cervix: a report of 34 cases of immunohistochemical and clinicopathological findings
Source: Front Oncol. 2026 Mar 2;16:1681399. doi: 10.3389/fonc.2026.1681399 (PMC12989362; doi:10.3389/fonc.2026.1681399)
Supplement: Supplementary file 2 [file Table2.docx]

Supplement table 2：Clinical characteristics of GAS

| ID | Age | FIGO Stage | LVSI | LNM | Maximum diameter(cm） | Invasion deep | PFS(days) | Outcom |
| --- | --- | --- | --- | --- | --- | --- | --- | --- |
| 1 | 38 | IV B | + | + | 5 | >2/3 | 351 | PD |
| 2 | 69 | IB3 | + | - | 4.5 | medium 1/3 | 671 | PR |
| 3 | 64 | IIIC1P | + | + | 5.7 | >2/3 | 367 | PD |
| 4 | 49 | IIIB | - | - | 6 | >2/3 | 260 | PD |
| 5 | 60 | IIIC1r | + | + | 4 | >2/3 | 185 | PD |
| 6 | 29 | IIIC1r | + | + | 6 | >2/3 | 171 | PD |
| 7 | 51 | IB3 | + | - | 3.5 | NA | 712 | PR |
| 8 | 47 | IV B | + | + | 5 | >2/3 | 178 | PD |
| 9 | 52 | IIIA | + | - | 5 | >2/3 | 307 | PD |
| 10 | 53 | IB3 | - | - | 4.5 | >2/3 | 571 | PR |
| 11 | 43 | IIA2 | - | - | 4.5 | >2/3 | 380 | PD |
| 12 | 37 | IIIB | + | - | 4.2 | >2/3 | 163 | PD |
| 13 | 65 | IIIC2P | + | + | 3.5 | >2/3 | 270 | PR |
| 14 | 53 | IB1 | - | - | 0.8 | medium 1/3 | 768 | PR |
| 15 | 36 | IB3 | + | - | 4.5 | >2/3 | 477 | PR |
| 16 | 50 | IB2 | + | - | 3 | >2/3 | 1508 | PR |
| 17 | 56 | IIIB | + | - | 3.3 | >2/3 | 483 | PD |
| 18 | 59 | IIIC1P | + | + | 2.5 | >2/3 | 136 | PD |
| 19 | 55 | IIIC1r | + | + | 6 | >2/3 | 554 | PR |
| 20 | 32 | IB2 | + | - | 3.8 | >2/3 | 465 | PR |
| 21 | 59 | IIIC1r | + | + | 3 | NA | 296 | PD |
| 22 | 31 | IIIC2P | + | + | 4 | >2/3 | 633 | PD |
