# Supplementary material for: Invasive stratified mucin-producing carcinoma of the cervix: a report of 34 cases of immunohistochemical and clinicopathological findings
Source: Front Oncol. 2026 Mar 2;16:1681399. doi: 10.3389/fonc.2026.1681399 (PMC12989362; doi:10.3389/fonc.2026.1681399)
Supplement: Supplementary file 3 [file Table3.docx]

Supplement Table 3

Multivariate Cox regression analysis of factors influencing patient progression-free survival

| Variable | No. of patients | Multivariate *P* value | Multivariate HR(95% CI) |
| --- | --- | --- | --- |
| Age |  | 0.248 | 0.509（0.162-1.602） |
| ＜45y | 49 |  |  |
| ≥45y | 55 |  |  |
| Tumor size |  | 0.412 | 1.616（0.513-5.089） |
| ＜3cm | 61 |  |  |
| ≥3cm | 43 |  |  |
| Histologic type |  | 0.008 | 0.218（0.071-0.667） |
| ISMC | 34 |  |  |
| HPV-A UEA | 70 |  |  |
| Initial FIGO stage |  | 0.584 | 1.781（0.226-9.036） |
| ＜I | 80 |  |  |
| ≥II | 24 |  |  |
| LVSI |  | 0.184 | 0.445（0.134-1.471） |
| no | 57 |  |  |
| present | 47 |  |  |
| LNM |  | 0.284 | 0.216（0.013-3.572） |
| no | 85 |  |  |
| present | 19 |  |  |
| Invasion depth |  | 0.302 | 0.575（0.202-1.642） |
| shallow1/3 | 38 |  |  |
| deep 2/3 | 66 |  |  |
| Silva pattern |  | 0.934 | 1.062（0.257-4.384） |
| A pr B | 32 |  |  |
| C | 72 |  |  |

COX proportional hazard regression model used for mualtivariable analysis.

*P*＜0.05 is considered statistically significant.
