# Supplementary material for: Invasive stratified mucin-producing carcinoma of the cervix: a report of 34 cases of immunohistochemical and clinicopathological findings
Source: Front Oncol. 2026 Mar 2;16:1681399. doi: 10.3389/fonc.2026.1681399 (PMC12989362; doi:10.3389/fonc.2026.1681399)
Supplement: Supplementary file 4 [file Table4.docx]

Supplement Table 4: Comparison of clinicopathological features between ISMC and GAS.

|  | ISMC (N =34) | GAS (N =22) | *P* value |
| --- | --- | --- | --- |
| Age，years | 42.4±9.4 | 49.5±11.5 | 0.021 |
| Maximum tumor diameter(cm) | 3.541±1.487 | 4.19±1.26 | 0.084 |
| Invasion depth |  |  | 0.145 |
| shallow1/3 | 5 | 0 |  |
| deep 2/3 | 29 | 20 |  |
| LVSI |  |  | 0.529 |
| No | 10 | 4 |  |
| Present | 24 | 18 |  |
| LNM |  |  | 0.752 |
| No | 20 | 12 |  |
| Present | 14 | 10 |  |
| FIGO stage 2018 |  |  | 0.17 |
| I stage | 18 | 7 |  |
| ≥ II stage | 16 | 15 |  |
